# Supplementary material for: Ciliated cells promote high infectious potential of influenza A virus through the efficient intracellular activation of hemagglutinin
Source: J Virol. 2025 Aug 29;99(9):e00685-25. doi: 10.1128/jvi.00685-25 (PMC12456007; doi:10.1128/jvi.00685-25)
Supplement: Supplemental figures — Figures S1 to S12. [file jvi.00685-25-s0001.pdf]

Supplementary Figures

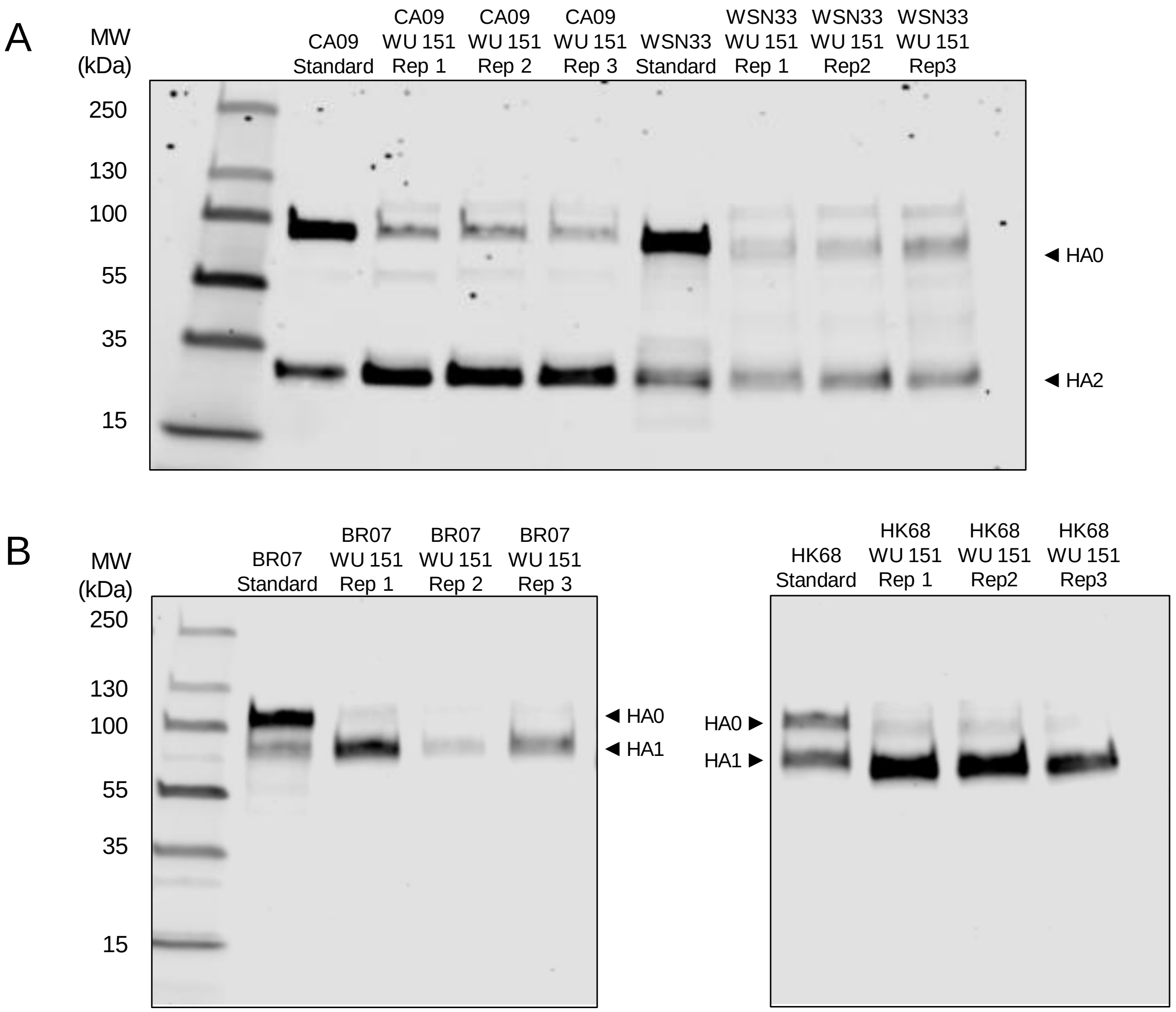

Supplementary Figure 1: Western blot replicates used to quantify HA activation in differentiated HTEC cultures. All experiments were performed using cells derived from a single donor(WU151).

(A) Results for H1 strains.

(B) Results for H3 strains. Blots are cropped to remove lanes not used in the experiment.

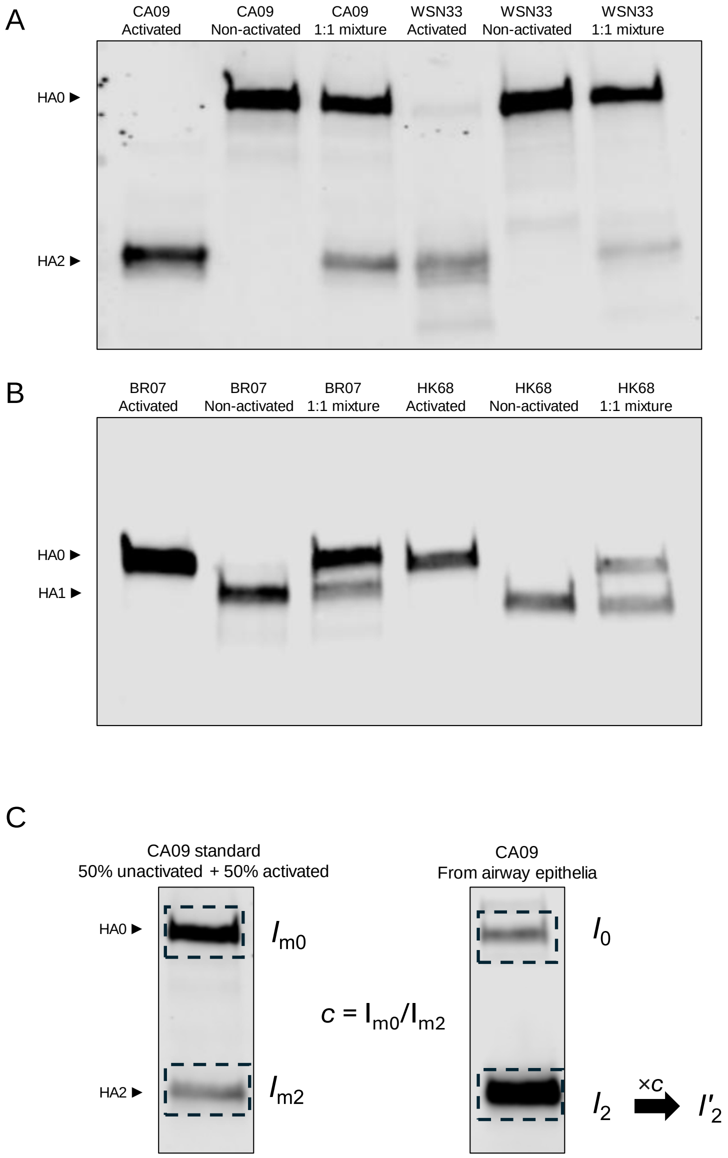

Supplementary Figure 2: Calibrating Western blot transfer efficiency using samples with equal amounts of unactivated and activated viruses.

(A) Western blot comparing fully-activated virus, unactivated virus, and a 1:1 mixture of activated and unactivated virus using H1N1 strains (CA09, WSN33) probed with antibody recognizing an epitope in HA2.

(B) Same as in *A*, but for H3N2 strains (BR07, HK68) probed with antibody recognizing an epitope in HA1.

(C) Determining the correction factor (‘*c*’) for activated / unactivated band intensities. The activation ratio is determined by the calibrated intensity of the activated band (I'_2) and the intensity of the

non-activated HA band (I_0).

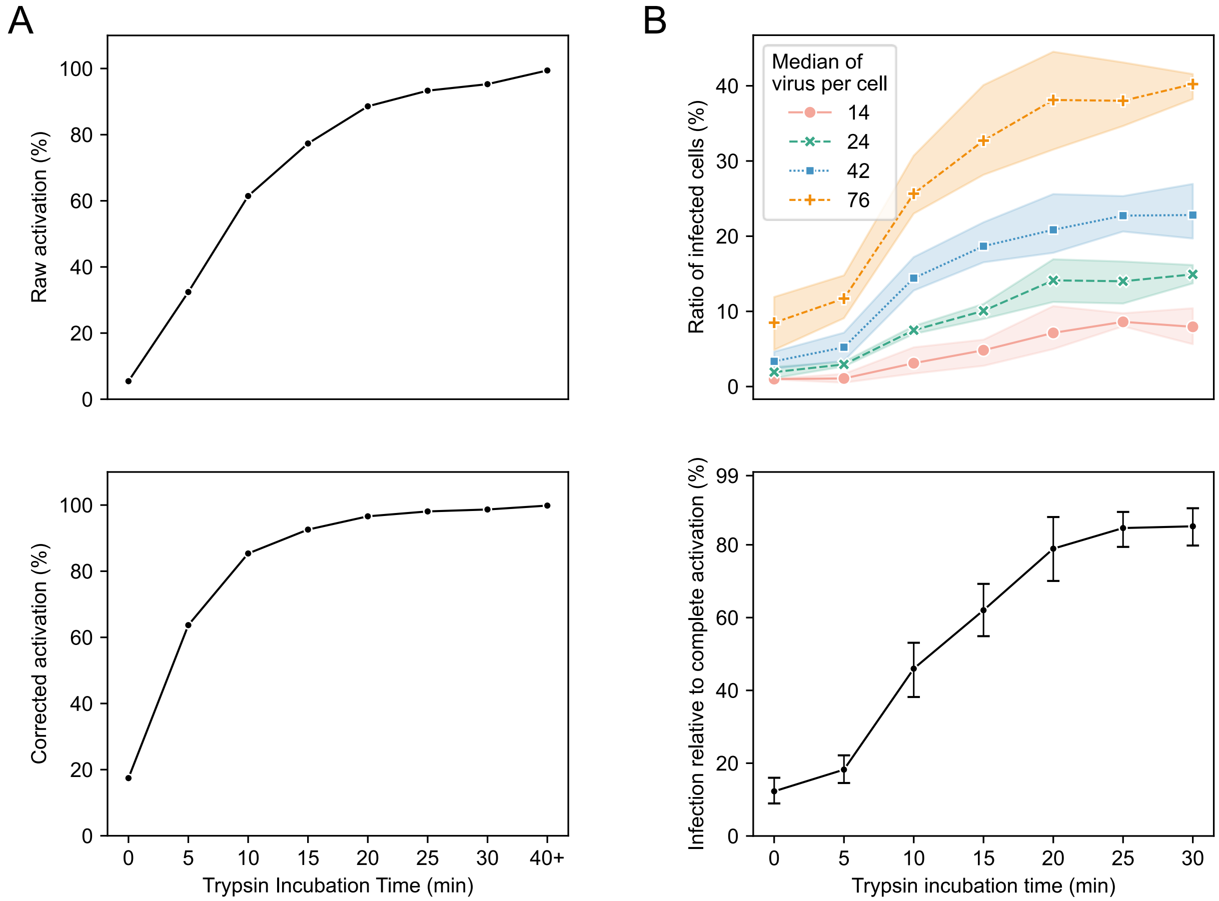

Supplementary Figure 3: Quantification of HA activation and infectivity of CA09.

(A) Quantification of HA activation for CA09 virus incubated with trypsin for different lengths of time. Data is from the Western blot in Figure 1E. Top: uncalibrated raw quantification of HA activation. Bottom: quantification accounting for different detection efficiencies of activated / unactivated bands.

(B) Top: Percentage of infected A549 cells following challenge with different amounts of virus, activated by trypsin for different amounts of time. Each curve shows experiments in which different amounts of virus was added and quantified based on the median number of virus per cell. Data is from three replicates. Bottom: Combined data from the top panel showing the relationship between percent infectivity and trypsin incubation time. Percent infectivity is determined relative to infection from samples with complete HA activation.

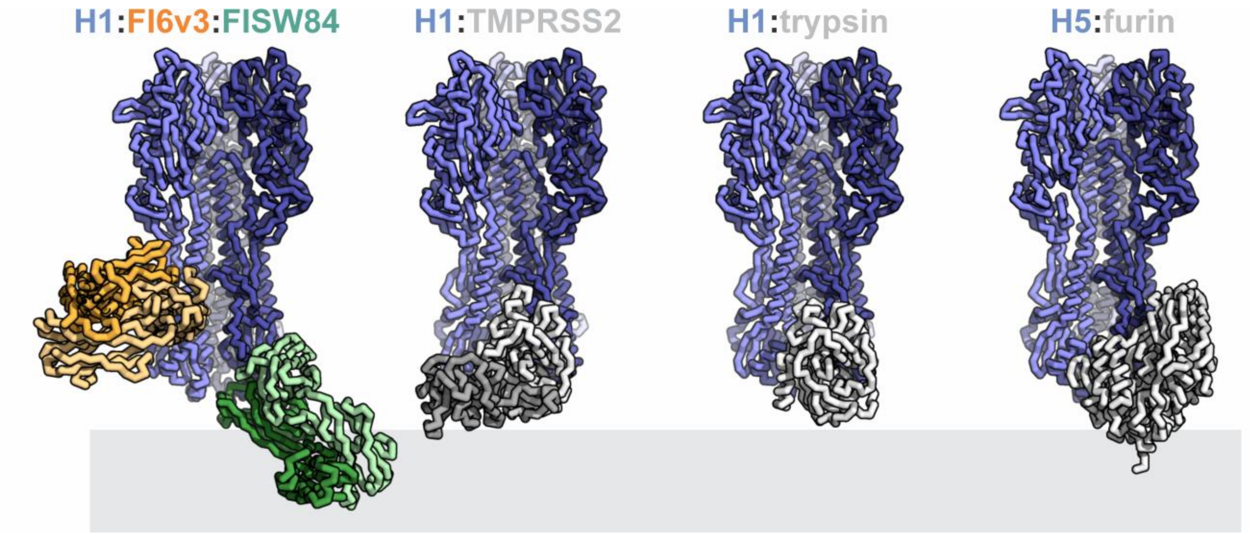

Supplementary Figure 4: AlphaFold2 models of serine proteases with HA predict steric clashes with stalk- and anchor-binding antibodies. Structures depict the unactivated HA trimer (blue) in complex with one of three proteases (human TMPRSS2, bovine trypsin, or human furin; shown in gray) or FI6v3 and FISW84 Fab fragments.

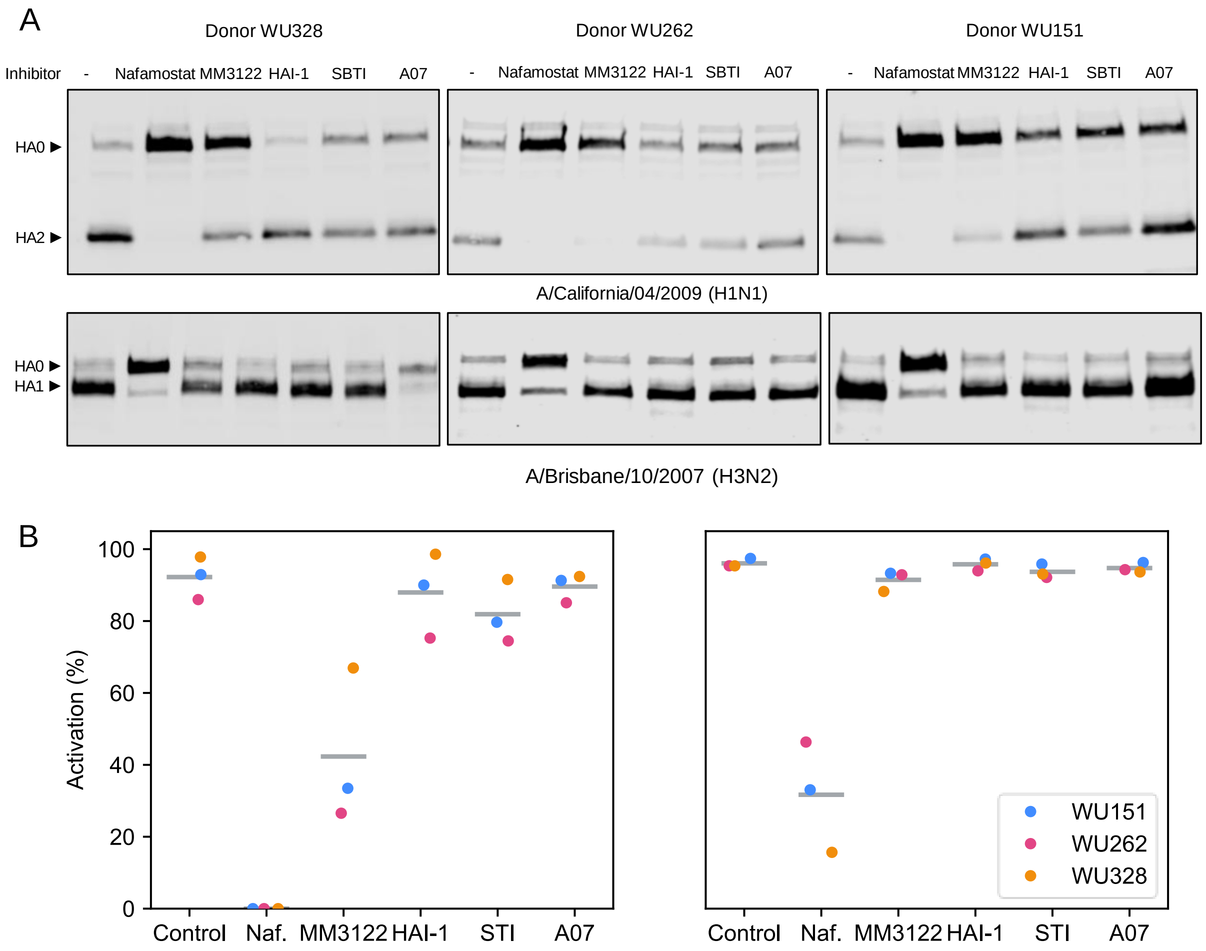

Supplementary Figure 5: Inhibition of HA activation in differentiated HTEC cultures by selected protease inhibitors.

(A) Western blots for quantifying HA activation inhibition of CA09 and BR07 strains by the indicated treatments. Differentiated cultures are derived from three donors.

(B) Quantification of Western blots shown in *A*. Values are calibrated based on differences in detection efficiencies between activated / unactivated bands.

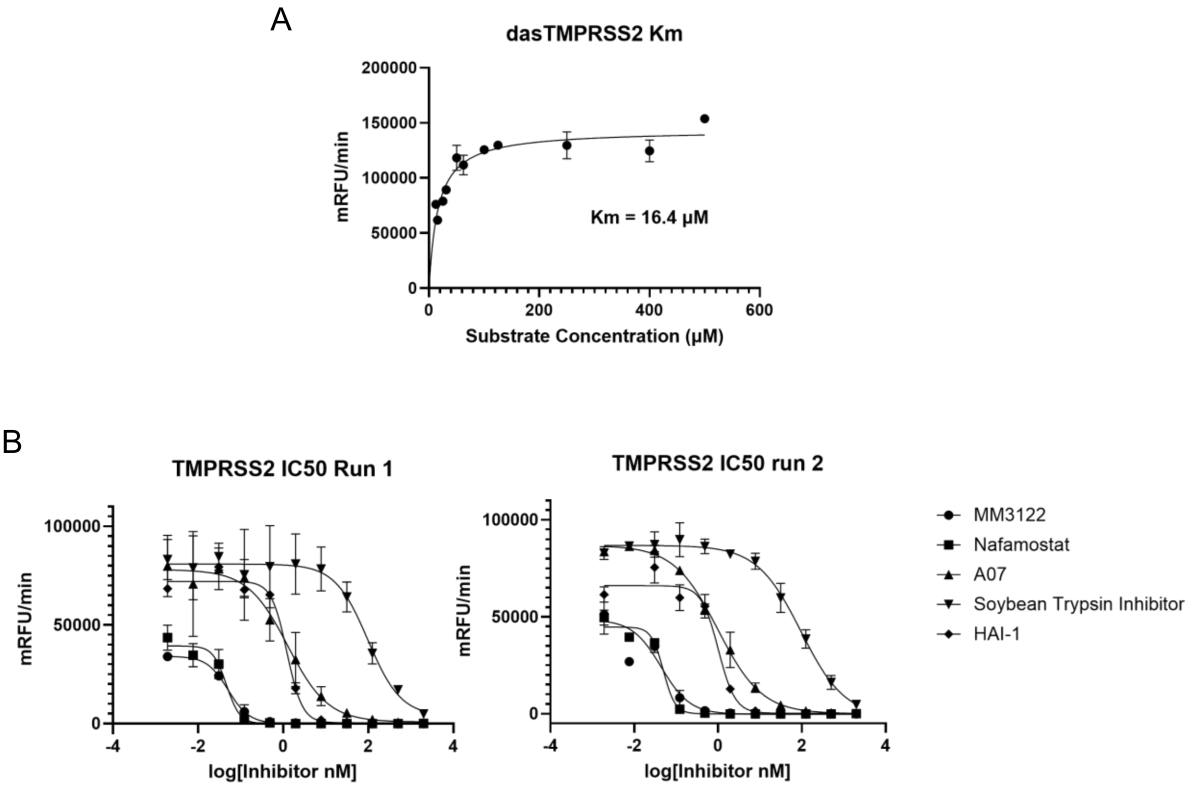

Supplementary Figure 6: IC50 values of inhibitors against recombinant TMPRSS2 ectodomain in a cell-free system.

(A) Michaelis-Menten plot of dasTMPRSS2, assayed at a final enzyme concentration of 3 nM. IC50 curves of tested compounds, assayed at a final enzyme concentration of 3 nM and final substrate concertation of 16 µM.

(B) IC50 curves of tested compounds, assayed at a final enzyme concentration of 3 nM and final substrate concertation of 16 µM.

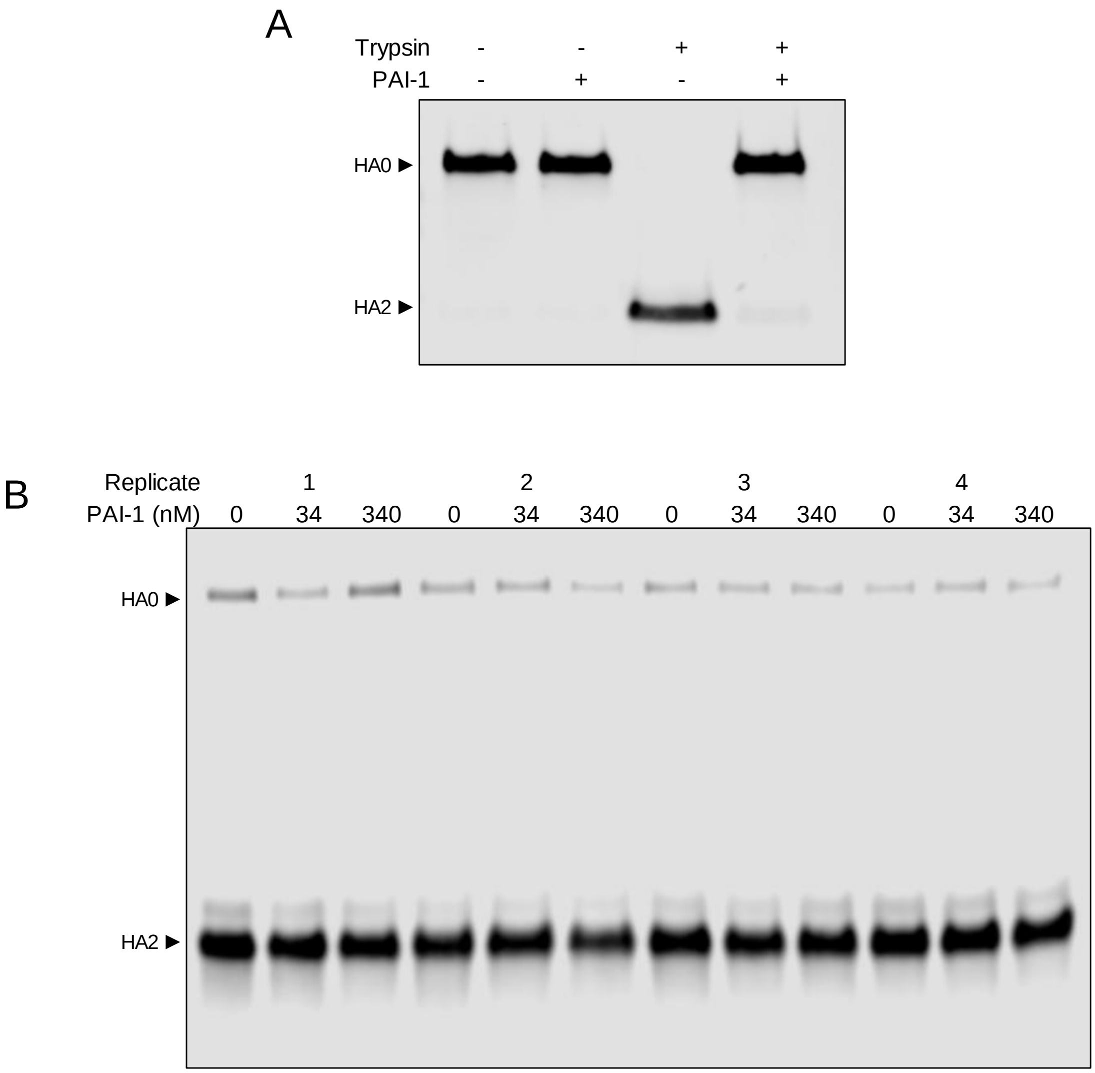

Supplementary Figure 7: Inhibition of HA activation in differentiated HTEC cultures using PAI-1.

(A) Western blot of inhibition tests in a cell-free context of 340 nM PAI-1 using CA09 (H1N1) virus produced by MDCK cells. TPCK-trypsin is used at 1 μg/mL (~40 nM).

(B) Western blot of HA from virus produced by differentiated HTEC cultures infected with CA09 in the presence of the indicated concentrations of PAI-1. Each lane represents virus from a single HTEC culture derived from the same donor.

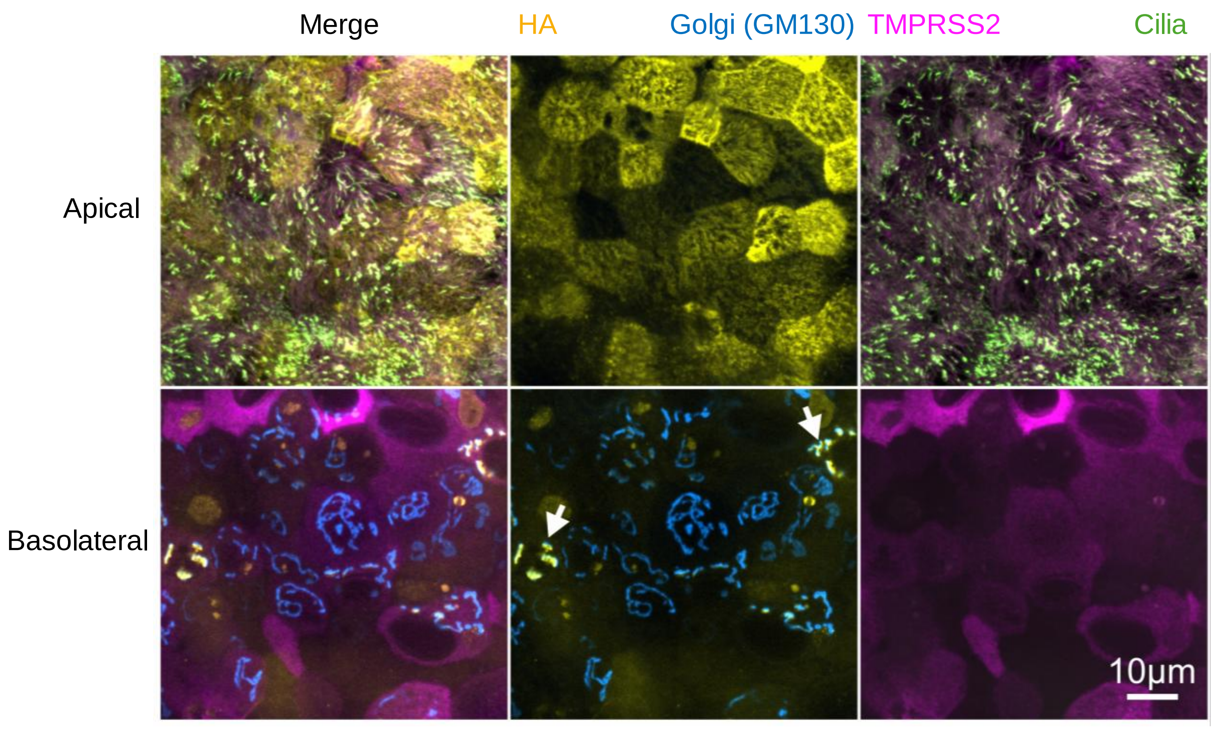

Supplementary Figure 8: Localization of HA and TMPRSS2 in the Golgi compartment. Immunofluorescence confocal images showing the expression of HA, the Golgi marker GM130, and TMPRSS2 in ciliated and non-ciliated cells in a differentiated HTEC culture infected by CA09. Images show apical and basolateral sections of the same field of view.

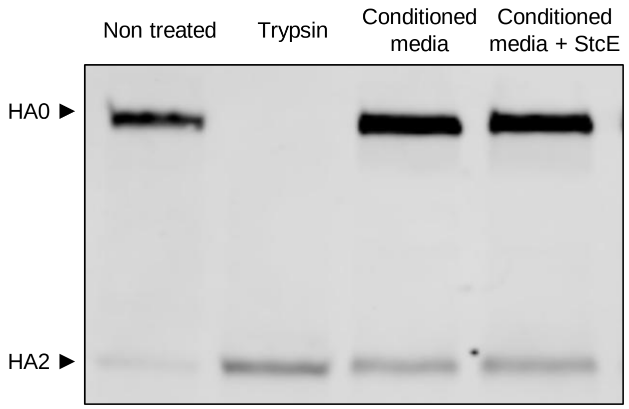

Supplementary Figure 9: Apical secretions from differentiated HTECs do not efficiently activate HA. Western blot of initially unactivated virus from MDCK cells following control treatment, treatment with trypsin, or treatment with apical secretions from differentiated HTEC cultures (+/- StcE). This experiment is performed with conditioned media from cells of donor WU151.

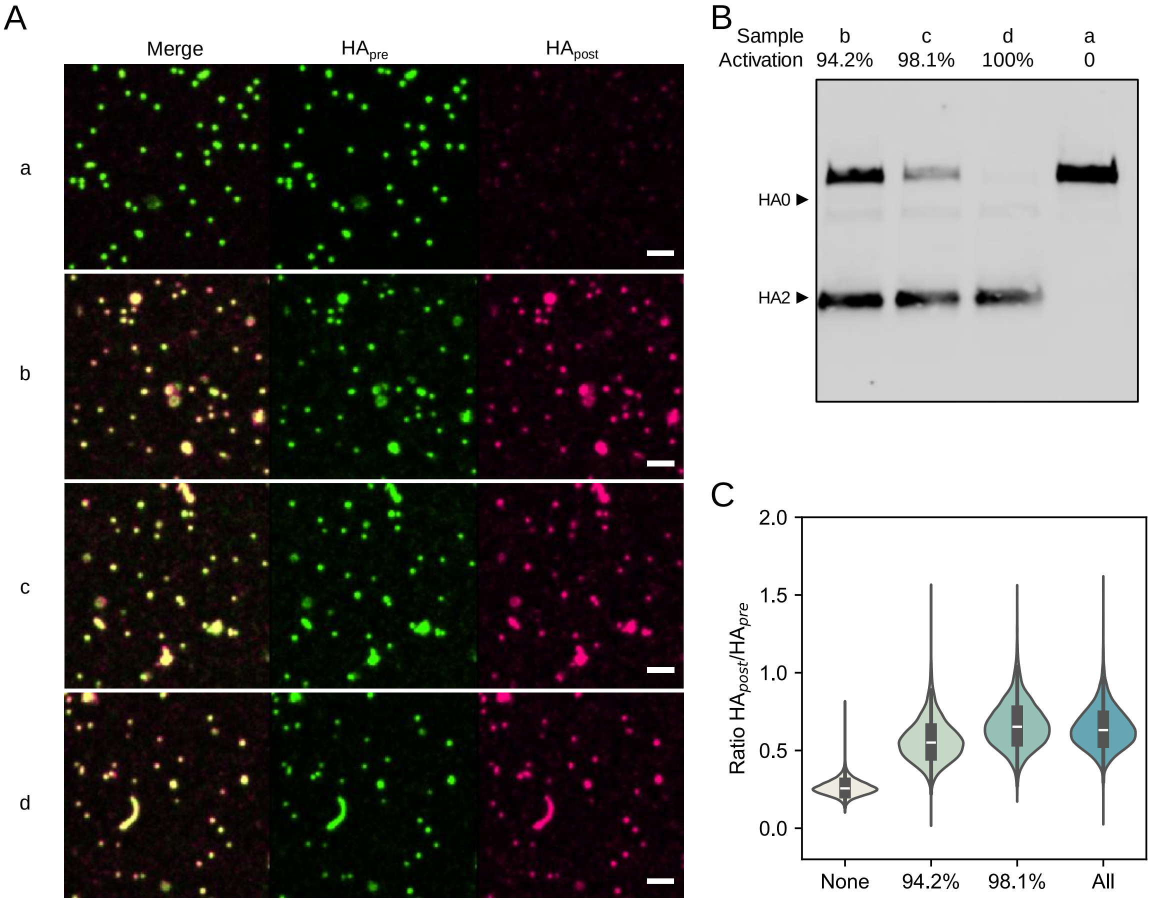

Supplementary Figure 10: Ratiometric labeling with pre- and post-fusion specific antibodies to quantify HA activation.

(A) Representative fluorescence images of viruses activated with trypsin for different amounts of time. Scale bar = 2 μm.

(B) Western blot of virus samples shown in *A*.

(C) The quantification of the ratio between HA_{post} and HA_{pre} from the fluorescence images. Data are pooled from single virion data from at least five fields of view containing a total of at least 10^4 virus particles.

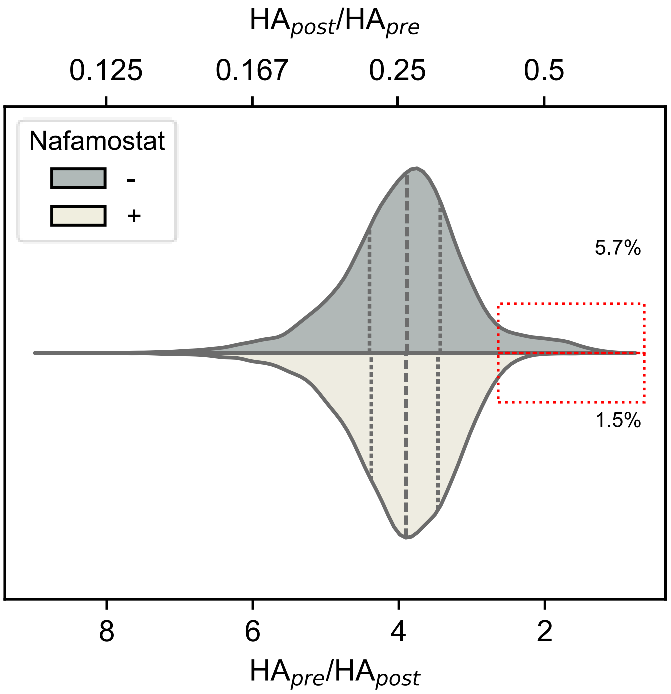

Supplementary Figure 11: Quantification of baseline HA activation of CA09 virus grown in MDCK cells. A small population of virions (red dashed box) show high activation in the absence of protease inhibitor. This population is suppressed in the presence of 100 nM Nafamostat.

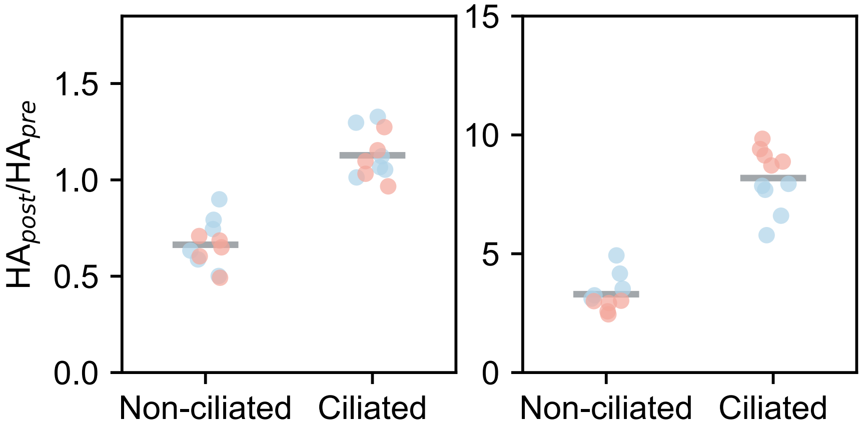

Supplementary Figure 12: Quantification of HA_{pre}/HA_{post} on non-ciliated and ciliated cells in differentiated HTECs infected by CA09 (left) and BR07 (right). Experiments were conducted on two independent samples from donor WU267, depicted by distinct colors in the plots. Each data point corresponds to a single field of view from immunofluorescence confocal imaging. Statistical tests are not available due to the limited number of donor cultures.
